# Post-COVID-19 Hyposmia Does Not Exhibit Main Neurodegeneration Markers in the Olfactory Pathway

**DOI:** 10.1007/s12035-024-04157-w

**Published:** 2024-04-04

**Authors:** Tommaso Schirinzi, Daniela Maftei, Riccardo Maurizi, Maria Albanese, Clara Simonetta, Roberta Bovenzi, Jacopo Bissacco, Davide Mascioli, Laura Boffa, Maria Grazia Di Certo, Francesca Gabanella, Beatrice Francavilla, Stefano Di Girolamo, Nicola Biagio Mercuri, Francesco Maria Passali, Roberta Lattanzi, Cinzia Severini

**Affiliations:** 1 Unit of Neurology, Department of Systems Medicine, Tor Vergata University of Rome, Via Montpellier 1, 00133 Rome, Italy; 2 Department of Physiology and Pharmacology “V. Erspamer”, Sapienza University of Rome, Rome, Italy; 3 Unit of ENT, Department of Clinical Sciences and Translational Medicine, Tor Vergata University of Rome, Rome, Italy; 4 Department of Biochemistry and Cell Biology, National Research Council of Italy, Rome, Italy

**Keywords:** Post-COVID, Long-COVID, Hyposmia, Olfactory dysfunction, Olfactory neurons, Neurodegeneration

## Abstract

The biological substrate of persistent post-COVID-19 hyposmia is still unclear. However, as many neurodegenerative diseases present with smell impairment at onset, it may theoretically reflect degeneration within the central olfactory circuits. However, no data still exist regarding the post-COVID-19 patients. As the olfactory neurons (ONs) mirror pathological changes in the brain, allowing for tracking the underlying molecular events, here, we performed a broad analysis of ONs from patients with persistent post-COVID-19 OD to identify traces of potential neurodegeneration. ONs were collected through the non-invasive brushing of the olfactory mucosa from ten patients with persistent post-COVID-19 hyposmia (lasting > 6 months after infection) and ten age/sex-matched controls. Immunofluorescence staining for protein quantification and RT-PCR for gene expression levels were combined to measure ONs markers of α-synuclein, amyloid-β, and tau pathology, axonal injury, and mitochondrial network. Patients and controls had similar ONs levels of oligomeric α-synuclein, amyloid-β peptide, tau protein, neurofilament light chain (NfL), cytochrome C oxidase subunit 3 (COX3), and the heat shock protein 60 (HSP60). Our findings thus did not provide evidence for synucleinopathy and amyloid-β mismetabolism or gross traces of neuronal injury and mitochondrial dysfunction within the olfactory system in the early phase of persistent post-COVID-19 hyposmia.

**Supplementary Information:**

The online version contains supplementary material available at 10.1007/s12035-024-04157-w.

## Introduction

Since the early phases of the outbreak, the risk for neurological sequelae of SARS-CoV-2 associate disease (COVID-19) has been hypothesized. Indeed, the virus may exert neuropathogenicity either directly, by entering into the CNS, or indirectly, through viral–host interactions and immune–inflammatory processes, inducing post-acute neurological syndromes or, eventually, triggering complex molecular events responsible for long-term consequences, such as neurodegenerative diseases [1].

The higher incidence of neurological disturbances shortly after the infection, within the so-called long-COVID, has been substantially demonstrated [2]. Conversely, the risk for neurodegenerative disorders cannot be clearly established yet. Recent studies identified some preliminary associations between COVID-19 and possible neurodegeneration; however, further unbiased investigations are definitely needed [3].

Persistent post-COVID-19 olfactory dysfunction (OD) consists of smell loss or impairment lasting several months (or years) after the acute infection and may affect up to 20% of COVID-19 patients [4, 5]. The pathophysiology mostly encompasses chronic inflammatory mechanisms in the olfactory pathway [6], associated with some possible rearrangements at a brain circuit level [7]. On the other side, hyposmia is a well-known prodromal symptom in Parkinson’s (PD), Alzheimer’s (AD), and other neurodegenerative diseases, which results from the early degeneration and dysfunction of the olfactory-limbic systems connections and other cortical-subcortical areas [8]. Accordingly, post-COVID-19 OD might theoretically represent an initial stage of a clinical-pathological trajectory leading to neurodegeneration [9].

Olfactory neurons (ONs) are the peripheral terminals of the olfactory system and, by the non-invasive olfactory mucosa brushing, can be collected to analyze in vivo the molecular events underlying the diseases. For example, it has been demonstrated that ONs accumulate neuropathological hallmarks of PD since the premotor phase, thus serving as a reliable model for central neurodegeneration [10, 11].

In this study, we examined ONs from patients with persistent post-COVID-19 OD for markers of main neurodegeneration pathways (α-synuclein, amyloid-β, tau, neurofilaments, mitochondrial network) in order to identify any cues supporting the risk for neurodegeneration in people with previous COVID-19.

## Materials and Methods

### Study Population

The study was conducted on a previously described cohort of ten patients with persistent post-COVID-19 OD and ten healthy sex/age-matched controls (CTRLs) enrolled at Tor Vergata University Hospital (Rome, Italy) in 2021–2022 [6] and subsequently observed up to July 2023, when they were clinically screened to exclude the occurrence of any cognitive/motor disorder consistent with neurodegenerative diseases.

Briefly, all patients had COVID-19 within November 2021 in Rome region, were suffering from OD since 6 to 10 months, and were SARS-CoV-2 negative at enrolment for 6 months at least. Controls were healthy volunteers without olfaction complaints, history, and clinical signs of neurological and otolaryngological diseases, never affected by COVID-19. Individuals with main acute/chronic infectious/inflammatory/internal diseases or under medications potentially interfering with data interpretation were excluded. For all subjects, demographics, anthropometrics, and medical history were collected. Olfaction was quantitatively assessed in patients by the “identification score” (IS) of the Sniffin’ Sticks test (Burghardt®, Wedel, Germany).

The study was approved by the local Ethical Committee (protocol n° 16.21), following the principles of Helsinki Declaration. All participants signed an informed consent.

### ONs Collection and Analysis

ONs were collected by olfactory mucosa brushing and processed as previously described [11]. The following markers were assessed by different techniques comparatively in patients and controls: oligomeric α-synuclein (α-syn) form, amyloid-β peptide (Aβ), tau protein, neurofilament light chain (NfL), cytochrome c oxidase subunit 3 (COX3), and the heat shock protein 60 (HSP60) (the latter’s as markers of mitochondrial activity).

### Immunofluorescence

Immunofluorescence analysis was performed on ONs previously fixed in Cytofix solution as reported [11]. Briefly, cells were cytocentrifuged onto microscope slides (Menzel Glaser, Superfrost® Plus), permeabilized in 0.2% Nonidet P-40 (Sigma-Aldrich) for 20 min, blocked in 5% normal donkey serum for 1 h, and incubated overnight at 4 °C with the following primary antibodies: rabbit anti-α-Syn33 (1:300; Millipore, CA) binding oligomeric α-syn; mouse anti-β-amyloid, which is directed against residues 1–16 of Aβ and also binds β-amyloid peptides (1:400; 6E10, BioLegend previously Covance); rabbit anti-tau (1:400; Sigma-Aldrich); mouse anti-OMP as the specific ONs marker-protein (1:300; Santa Cruz Biotechnology); rabbit anti-β3-tubulin, as a neuronal marker (TU-20; 1:300; Cell Signaling Technology); mouse anti-HSP60 (H-1; 1:100; Santa Cruz Biotechnology).

Slides were then washed with PBS and incubated for 1 h at room temperature, with anti-species IgG secondary antibodies coupled to Alexa Fluor 488 or 555 (Immunological Sciences). Nuclei were stained with DAPI (Sigma-Aldrich). The fluorescent signal was acquired by an Eclipse E600 fluorescence microscope (Nikon Instruments, Japan) connected to a QImaging camera with NIS-Elements BR 3.2 64-bit software.

To quantify the immunofluorescence intensity, images were acquired at high magnification with a × 100 or × 60 objective maintaining exposure parameters, such as gain and time, constant to avoid observing differences between experimental groups due to artifacts. The RGB fluorescent signal was automatically analyzed using ImageJ software (version 1.53, National Institutes of Health, USA; https://imagej.nih.gov/ij/download.html) and RGB (red, green, blue) measure tool, which uses brightness values for the calculation. A number of 5–6 images were taken from each sample and an average was obtained. Unit of measurements is pixel size predetermined by the ImageJ software.

### RNA Extraction and Real-Time PCR

Total RNA was extracted from ONs using TRIzol reagent and processed as previously described [11].

Briefly, 1 µg of total RNA was reverse transcribed into cDNA (Reverse Transcriptase, Bioline Meridian Bioscience) and then amplified by real-time PCR (iCycler; Bio-Rad) using iQ SYBR Green Supermix (Bioline Meridian Bioscience) and specific sense and antisense human primers targeting NfL and mitochondrial gene COX3 (Eurofins Genomics, Ebersberg, Germany). All reactions were run in triplicate under the same thermal cycling conditions, and the average was normalized to the reference gene GAPDH. Gene expression was analyzed using the comparative (2^−ΔΔCt^) method, and results were presented as a fold increase of the target gene compared to the control group.

Primer sequences:

- NfL Fw 5′-CAAGACCCTGGAAATCGAAG-3′, Rev 5′-TGAAACTGAGTCGGGTCTCC-3′
- COX3 Fw 5′-ATGACCCACCAATCACATGC-3′, Rev 5′-ATCACATGGCTAGGCCGGAG-3′
- GAPDH Fw: 5′-TGCACCACCAACTGCTTAGC-3′, Rev: 5′-GGCATGGACTGTGG TCATGAG-3′.

### Statistical Analysis

Variable distribution was preliminarily examined by the Shapiro–Wilk test. Non-normally distributed variables were Log_10+1_ transformed when necessary for analysis. Categorical variables were compared by chi-square test, the continuous ones instead by parametric (Student’s *T*-test) or non-parametric tests, as appropriate. Statistical significance was set at *p* < 0.05. Analysis was run in blind, by using IBM-SPSS-23 and GraphPad Prism 7.

## Results

### Study Population

The post-COVID-19 OD group included seven females and three males with mean ± st.dev age of 43 ± 13 years. The control group included six females and four males with an age of 50 ± 14 years. Sex and age did not differ between the groups. The Sniffin’ Sticks test IS was significantly lower in patients (10.5 ± 3.2) than in controls (15.6 ± 0.7, *U* = 2.5, *p* < 0.001). Further details were previously published [6] and available in a supplementary Table for convenience.

### Evaluation of Neurodegeneration-Related Markers

**Fig. 1:**
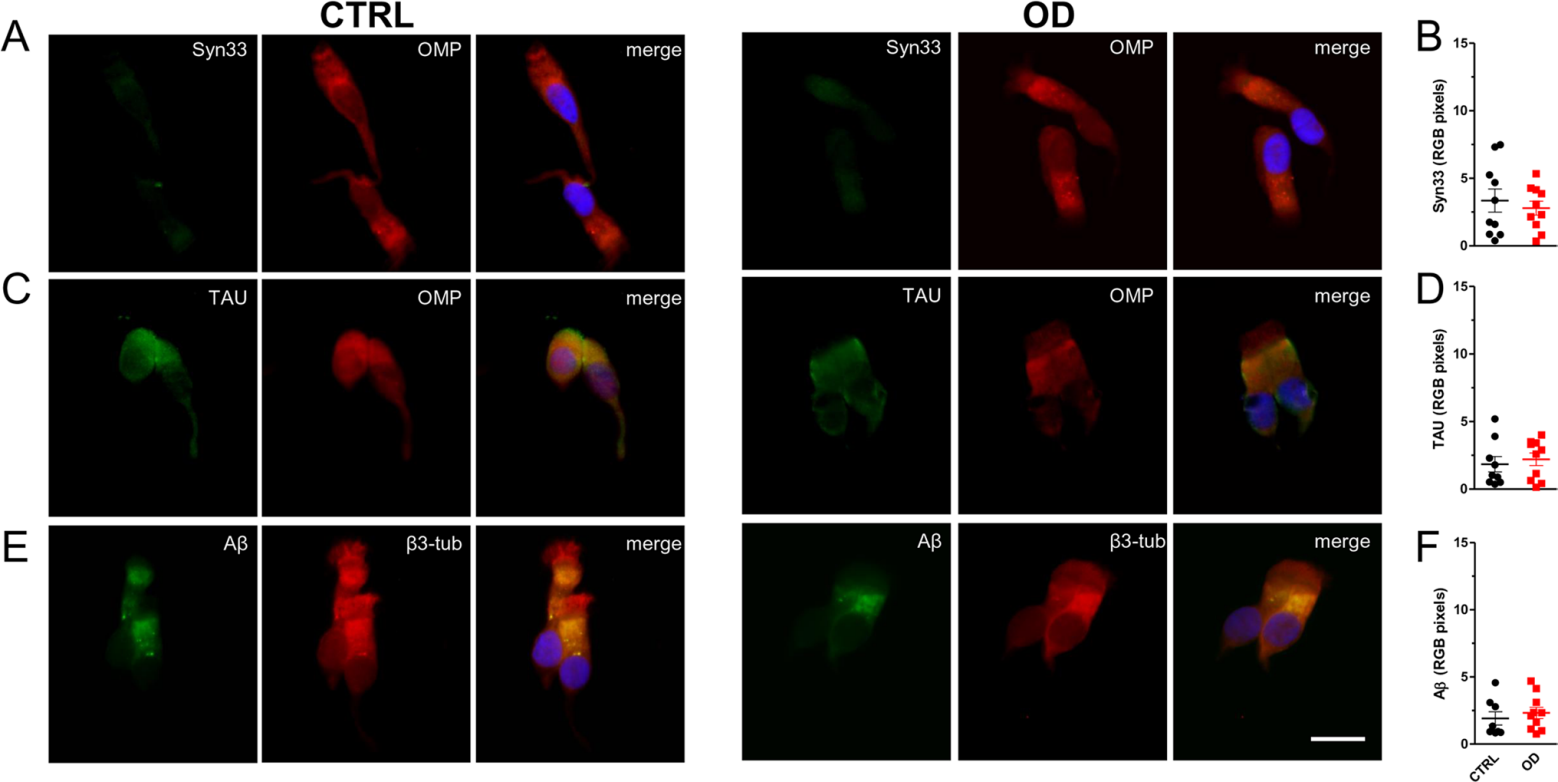
Neurodegeneration-associated marker expression levels. Representative immunofluorescent images showing the expression pattern of oligomeric α-syn (green) (**A**), tau protein (green) (**C**) and Aβ (green) (**E**) in ONs from healthy controls (CTRL) and post-COVID-19 OD patients. Nuclei were stained with DAPI (blue). Scale bar, 10 µm. Graphs showing immunofluorescence signal quantification of oligomeric α-syn (**B**), tau protein (**D**), and Aβ (**F**) in each group evaluated by ImageJ software. Data points represent the mean value ± SEM

The double immunostaining of oligomeric α-syn and OMP showed a similar immunofluorescence signal in patients and controls (Fig. 1A), as further confirmed by immunofluorescence analysis (RGB pixels analysis): OD patients (*n* = 10, 2.8 ± 0.51) versus CTRLs (*n* = 10, 3.3 ± 0.85) (Fig. 1B).

As well, the double immunofluorescence staining of tau with OMP and Aβ with β3-tub excluded significant differences between patients and controls (Fig. 1C, E), as demonstrated by RGB immunofluorescence analysis: Tau, OD (*n* = 10, 2.2 ± 0.46) versus CTRLs (*n* = 9, 1.8 ± 0.56; Aβ, OD (*n* = 10, 2.3 ± 0.41) versus CTRLs (*n* = 8, 1.9 ± 0.49) (Fig. 1D, F). The immunofluorescence expression patterns observed for tau and Aβ were consistent with previous data reported by Brozzetti and colleagues [10]. Tau positivity presented a homogenous intracytoplasmic distribution, while Aβ positivity showed a dot-like positivity, distributed in the proximity of the nucleus.

**Fig. 2:**
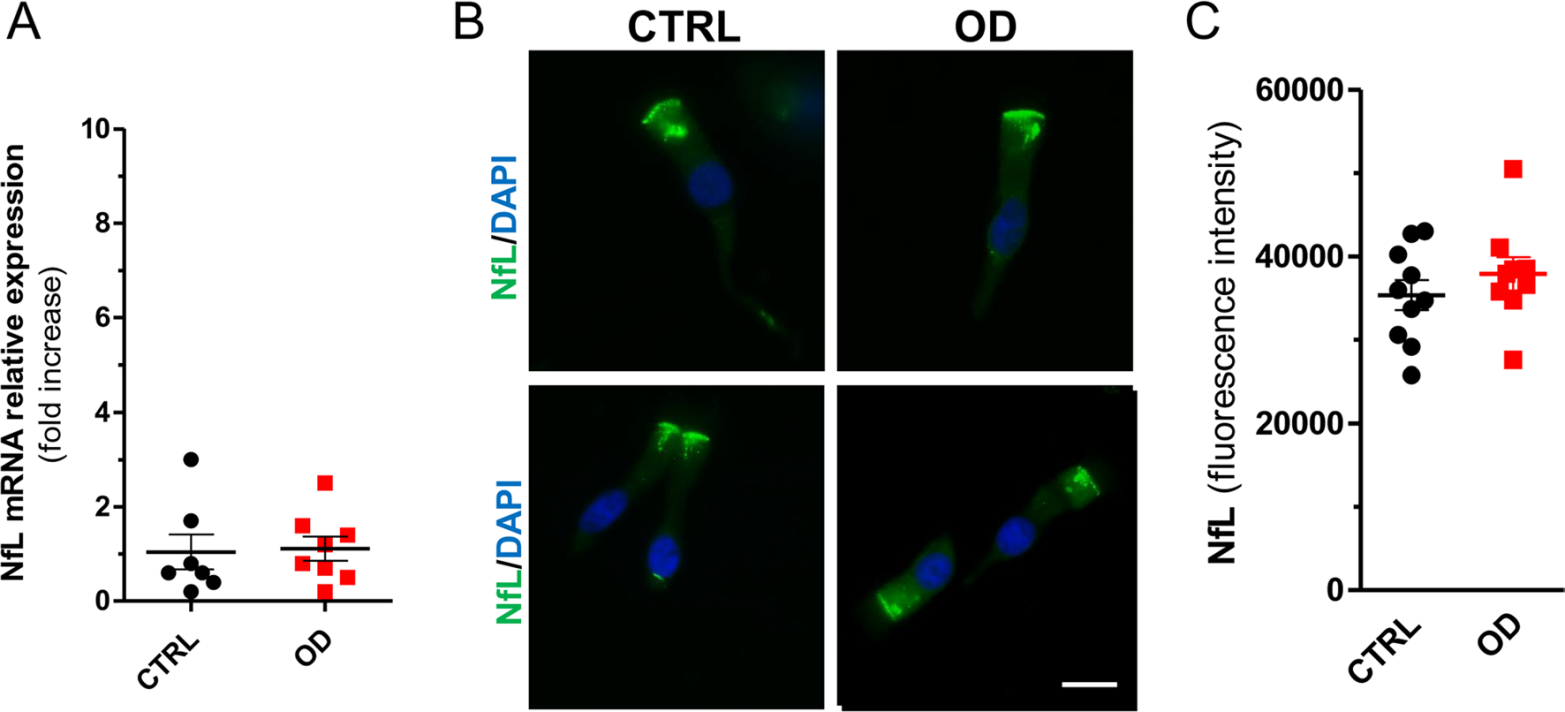
Neurofilament light chain (NfL) expression levels. **A** The NfL mRNA expression level in ONs from post-COVID-19 OD patients and controls. **B** Representative immunofluorescence analysis of NfL protein (green) in ONs. Nuclei were stained with DAPI (blue). Scale bar, 10 µm. **C** Graphs showing immunofluorescence intensity of NfL staining in each group evaluated by ImageJ software. Data points represent the mean value ± SEM

NfL mRNA expression levels were similar in patients and controls: OD (*n* = 8, 1.11 ± 0.25) versus CTRLs (*n* = 7, 1.04 ± 0.37) (Fig. 2A). As well, the NfL protein levels measured through the immunofluorescence assay did not differ: OD (*n* = 9, 37,930 ± 2012) versus CTRLs (*n* = 10, 35,380 ± 1819) (Fig. 2B, C). The fluorescent signal of NfL localized in the cytoplasm of ONs (Fig. 2B). No intersex differences resulted in patients and controls.

### Evaluation of Mitochondrial Network

**Fig. 3:**
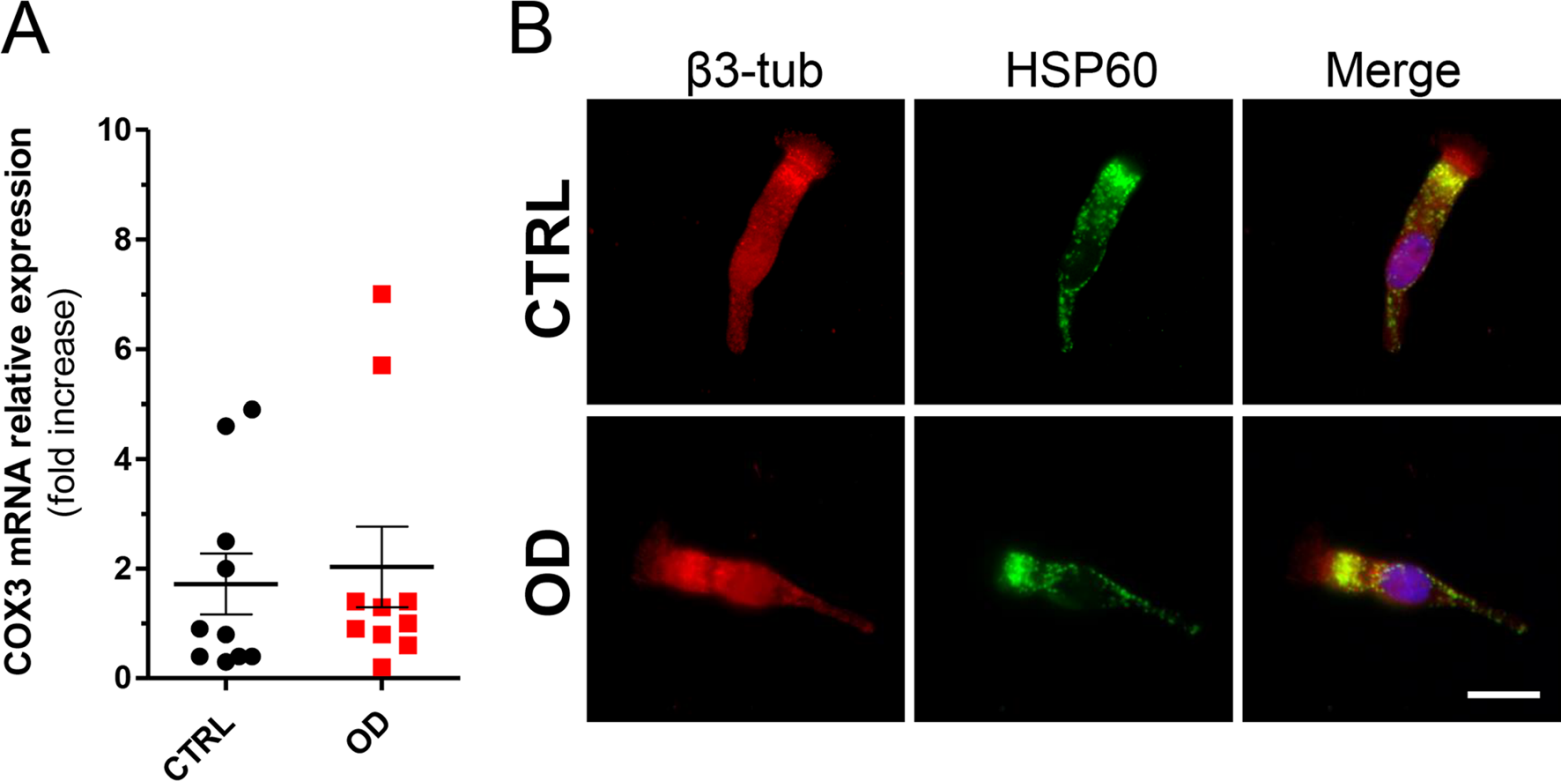
Mitochondrial network markers. **A** COX3 mRNA expression levels in ONs from post-COVID-19 OD patients and controls. Data points represent the mean value ± SEM. **B** Representative immunofluorescence analysis of the mitochondrial protein HSP60 (green) with the β3-tubulin (red) and in ONs. Nuclei were stained with DAPI (blue). Scale bar, 10 µm

COX3 mRNA expression levels were similar in patients and controls: OD (*n* = 10, 2.03 ± 0.73) versus CTRL (*n* = 10, 1.72 ± 0.55) (Fig. 3A). As well, the HSP60 localization pattern analyzed by immunofluorescence did not differ. The characterization of the mitochondrial reticulum of the neuronal cells, identified by the β3-tub staining, exhibited a similar pattern between patients and controls, without significant changes in morphology and subcellular distribution (Fig. 3B).

## Discussion

The olfactory system is critical in pathogenic trajectories underlying neurodegenerative diseases. In particular, some authors hypothesized that the olfactory route may serve as an entry site for external factors (viruses or toxicants) triggering synucleinopathy in PD [12]. As well, it results as early affected by tau and amyloid-β pathology in AD [13, 14]. In parallel with these pathological changes, also the olfactory function declines such that hyposmia may long precede classical manifestations of PD and AD, representing a prodromal marker or a readout for pathological progression in these conditions [9, 13].

Theoretically, even persistent post-COVID-19 OD might thus origin from the impairment of the olfactory system, which can imply a simple signaling dysfunction or cell loss with neurodegeneration; however, no definitive data still exist. Recent studies showed that peripheral ONs may express some of the neurodegenerative diseases’ biological hallmarks, thus emerging as a potential model to assess molecular stages of neurodegeneration in vivo [10, 11]. Accordingly, here, we performed a broad characterization of ONs from patients with persistent post-COVID-19 hyposmia to ascertain (or exclude) the presence of neurodegeneration-associated markers, including synucleinopathy, amyloid-β mismetabolism, neuronal injury, and mitochondrial dysfunction. Specifically, we assessed ONs for oligomeric α-synuclein, a pathological α-synuclein specie characterizing PD neuropathology and accumulating in PD patients [11]; amyloid-β peptide, one of the core determinants of AD pathology [15], which can locate in the olfactory epithelium either in patients or in animal models [16, 17]; tau protein, a cytoskeletal component associated with neuronal loss and AD pathology that can settle within the olfactory system of AD patients [18]; NfL, a marker of non-specific neuroaxonal injury [19]; COX3, a mitochondrial respiratory chain enzyme [20]: and HSP60 [21], both serving as readouts of the mitochondrial function. Of relevance, we found that none of these markers was differently expressed in patients and healthy controls, excluding the pathological activation of these pathways associated with main neurodegenerative diseases in symptomatic hyposmic post-COVID-19 patients during the first period after the infection.

The pathophysiology of persistent post-COVID-19 hyposmia is not clearly understood yet. In a previous paper similarly analyzing ONs, we demonstrated that the substance P (SP) overexpression, consistent with a long-lasting inflammation within the olfactory system, was contributing to smell impairment; conversely, prokineticin-2 (PK2), an inducible inflammatory mediator exerting neuroprotective and olfactogenesis functions, was mitigating or supporting the recovery [6].

SP and PK2 might play the same differential roles even in PD pathogenesis (and neurodegeneration at all). In fact, in ONs from PD patients, SP levels rise proportionally with the clinical severity, operating as a pathogenic force [22–24], while PK2 expression follows oligomeric α-synuclein accumulation, providing a sort of defensive response [11, 25, 26].

Merging these data together, we could thus hypothesize that, in the early phase of persistent post-COVID-19 hyposmia (e.g., up to 1 year from the onset), the olfactory system only exhibits inflammatory events but not overt proteinopathy-related neurodegeneration. Then, the individual capacity to resolve such inflammation, as the occurrence of dual activation of pro- and anti-inflammatory pathways (SP and PK2, respectively) suggests, determines the possibility of future neurodegeneration.

This study has some limitations, including the sample size and the exiguity of the neurodegeneration-associated marker panel, which indeed prevents a wider assessment of different pathological cascades. Moreover, an integration with measuring marker levels (especially NfL [19]) in fluids (serum) might have better reflected ongoing processes. Nevertheless, we showed no gross traces of the main neurodegenerative cascades, especially those related to proteinopathy, in the olfactory system of patients with persistent post-COVID-19 hyposmia within the first year from the onset. Further observation is thus needed to assess the possibility of neurodegeneration as a long-term consequence of COVID-19.

## Supplementary Information

Below is the link to the electronic supplementary material.

Supplementary file1 (DOCX 15 KB)

## Data Availability

Data are provided within the manuscript or by the author under reasonable request.
