# Supplementary material for: Post-COVID-19 Hyposmia Does Not Exhibit Main Neurodegeneration Markers in the Olfactory Pathway
Source: Mol Neurobiol. 2024 Apr 4;61(11):8921–7. doi: 10.1007/s12035-024-04157-w (PMC11496343; doi:10.1007/s12035-024-04157-w)
Supplement: Supplementary file 1 — Supplementary file1 (DOCX 15 KB) [file 12035_2024_4157_MOESM1_ESM.docx]

**Supplementary Table.**

**Summary of main clinical and neuroimaging findings of post-COVID-19 OD patients.** *(Published in Schirinzi T, et al., Brain Behav Immun. 2022;108:302–8 – Permission obtained from the authors).*

| **Patient** | **Age** | **Sex** | **Other neurological complaints** | **Brain MRI** |
| --- | --- | --- | --- | --- |
| 1 | 29 | F | No | NP |
| 2 | 57 | F | No | NP |
| 3 | 47 | F | No | NP |
| 4 | 29 | F | Transient insomnia | NP |
| 5 | 61 | F | Persisting depression | Minimal periventricular gliosis |
| 6 | 50 | F | Transient headache | Normal |
| 7 | 30 | F | Transient headache | Normal |
| 8 | 33 | M | No | NP |
| 9 | 36 | M | No | Normal |
| 10 | 58 | M | No | NP |

F = female, M = male, NP = not performed, age is expressed in years.
